# Ultrashort Echo Time and Fast Field Echo Imaging for Spine Bone Imaging with Application in Spondylolysis Evaluation

**DOI:** 10.3390/computation12080152

**Published:** 2024-07-24

**Authors:** Diana Vucevic, Vadim Malis, Yuichi Yamashita, Anya Mesa, Tomosuke Yamaguchi, Suraj Achar, Mitsue Miyazaki, Won C. Bae

**Affiliations:** 1 Dept. of Radiology, University of California-San Diego, San Diego, CA 92103; 2 Canon Medical Systems Corporation, Tochigi, Japan; 3 Dept. of Family Medicine, University of California-San Diego, San Diego, CA 92103, USA; 4 Department of Radiology, VA San Diego Healthcare System, San Diego, CA 92161, USA

**Keywords:** MRI, Low Back Pain, Bone Fracture, Pars Interarticularis

## Abstract

Isthmic spondylolysis is characterized by a stress injury to the pars interarticularis bones of the lumbar spines, often missed by conventional magnetic resonance imaging (MRI) necessitating a computed tomography (CT) for accurate diagnosis. We compare MRI techniques suitable for producing CT-like images. Lumbar spines of asymptomatic and low back pain (LBP) subjects were imaged at 3-Tesla with multi-echo ultrashort echo time (UTE) and field echo (FE) sequences followed by simple post-processing of averaging and inverting to depict spinal bone with CT-like appearance. Contrast-to-noise ratio (CNR) for bone was determined to compare UTE vs. FE and single-echo vs. multi-echo images. Visually, both sequences depicted cortical bone with good contrast; UTE-processed provided a flatter contrast for soft tissues that made it easy to distinguish from bone, while FE-processed images had better resolution and bone-muscle contrast, important for fracture detection. Additionally, multiecho images provided significantly (p=0.03) greater CNR compared single-echo. Using these techniques, a progressive spondylolysis was detected in a LBP subject. This study has demonstrated the feasibility of spine bone MRI to yield CT-like contrast. Through the employment of multiecho UTE and FE sequences combined with simple processing, we have observed enhancements in image quality and contrast, sufficient to detect pars fracture.

## 1. Introduction

Low back pain (LBP) affects a significant proportion of young athletes, with up to 36% reporting it annually [1]. Isthmic spondylolysis [2] is characterized by a stress injury to the pars interarticularis of the vertebral arch of the lumbar spine [3,4]. It is a prevalent condition among young athletes engaged in activities that place repetitive stress on the lumbar spine. While the exact incidence of isthmic spondylolysis is not well-documented, studies have reported it as a frequent cause of low back pain in young athletes, with prevalence rates ranging from 20% to 50% [5]. These findings highlight the need for accurate, radiation-free diagnostic tools for young individuals.

Isthmic spondylolysis presents with varying severity, necessitating different imaging techniques for accurate assessment. In the initial stage of stress injuries to the pars interarticularis characterized by bone marrow edema without fracture, magnetic resonance imaging (MRI) is the most sensitive tool for detecting bone stress reaction [6,7]. As the condition progresses, both bone stress reaction and fracture may be present, while the terminal stage involves only fracture. Computed tomography (CT) is superior for visualizing bone fractures. This variability in presentation makes it challenging for a single imaging modality to fully characterize the stage of isthmic spondylolysis. Both MRI and CT play crucial roles in the evaluation, and frequently both modalities are necessary for a definitive diagnosis.

Due to concerns about ionizing radiation exposure in young patients, particularly those of reproductive age, MRI is the preferred initial imaging modality for detecting isthmic spondylolysis. However, due to limited ability to visualize bone detail conventional MRI frequently fails to identify a significant number of bone fractures that are later detected by CT scans [6–8]. A follow-up CT scan, providing excellent contrast and visualization for fine bone details [9], is then needed to ascertain the diagnosis for bone fracture at the pars. While multiple CT scans are detrimental for long-term health of adolescent subjects, increasing the risk of cancer [10–12], it can be unavoidable in those who sustain recurrent low back injury, known to occur in as much as 33% of sport back injury patients within a year [13]. Reduction or avoidance of radiation would be highly beneficial for imaging of young athletes suffering from low back pain.

Newer MRI techniques are gradually being adapted for bone imaging with the development of sequences like ultrashort echo time (UTE) [14], and zero echo time (ZTE) [15]. These techniques have shown potential in visualizing bone structure in several joints including the knee [15,16], shoulder [17], and the lumbar spine [18]. Nonetheless, they are hampered by inherent limitations such as a low signal-to-noise ratio (SNR) and lower resolution compared to CT. Continued study and development of bone-oriented MR techniques is warranted.

In this study, our objective was to introduce and compare two MRI techniques for imaging bone: multiecho UTE and multiecho field echo (FE) acquisitions combined with simple post-processing to enhance appearance, SNR, and image contrast, aiming to reach CT in terms of image characteristics. The use of multi-echo images to improve SNR has been proposed previously [19,20] and it will likely benefit UTE imaging for depicting cortical bone. FE sequences have also been suggested for bone imaging [21], with notable strengths including the wide availability of the sequence from major MRI vendors, high spatial resolution, and high contrast between muscle and bone, useful for bone visualization. In this study, we combined UTE and FE acquisitions with simple post-processing techniques to provide CT-like appearance and improve image quality.

## 2. Materials and Methods

### 2.1. Subjects

Lumbar spines of four subjects (n=4, 3 females, 19.5 ± 5.5 years old) were imaged at 3-T (Canon Galan) with a posterior spine coil. Two health volunteers were recruited with the inclusion criteria of no low back pain or a history of low back surgery. Two of the subjects were adolescent (16 yo female, 14 yo male) athletes with non-specific and non-radiating low back pain for the past 4–8 weeks. The symptoms did not improve with conservative treatment, and the patients’ primary care physician referred them to be evaluated for the presence of spondylolysis.

### 2.2. MRI

**Figure 1.**
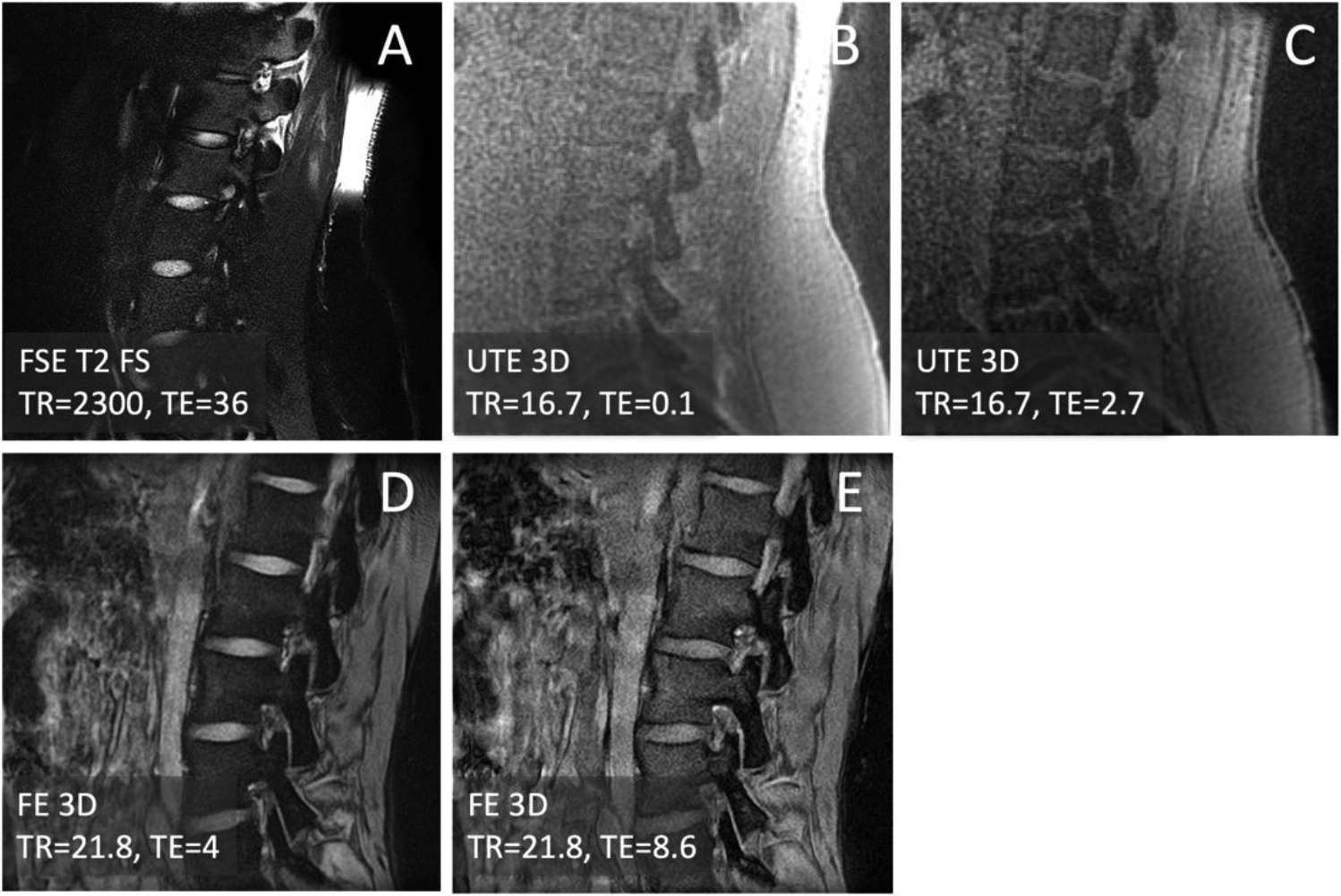
Raw MRI images of a lumbar spine acquired with (A) fast spin echo T2 weighted fat suppressed (FSE T2 FS), (B, C) 3D ultrashort echo time (UTE) at varying echo times (TE), and (D, E) 3D field echo (FE) at varying TE.

The subjects were imaged with a 3-Tesla MRI scanner (Galan, Canon Medical Systems Corp., Otawara, Japan) fitted with a posterior receive-only spine coil. One anatomical sequence and two bone-oriented sequences were used to image each subject. The anatomical sequence was a sagittal fast spin echo T2 weighted (**FSE T2**) with fat suppression with the scanning parameters of: repetition time (TR)= 5,600 milliseconds (ms), echo time (TE)=80 ms, field of view (FOV)=220 millimeters (mm), image matrix=320×416, slice thickness=3 mm, echo train length (ETL)=19. The bone-oriented sequences were: an axial 3D **UTE** multi-echo sequence with scan parameters of: TR=16.7 ms, TE=0.1, 2.7, 5.3 ms, number of projections=19968, FOV=300 ms, matrix=320×320, slice=1 mm, reformatted to sagittal plane; and a sagittal 3D field echo (**FE**) multi-echo sequence with scan parameters of: TR=21.8 ms, TE=4, 8.6, 13.2 ms, FOV=170 mm, matrix=320×240, slice=1 mm. The source images are shown in Figure 1. While no formal optimization was performed, we initially tried different numbers of radial projections for 3D UTE, and different TE values for 3D FE.

### 2.3. CT-like Image Processing

**Figure 2.**
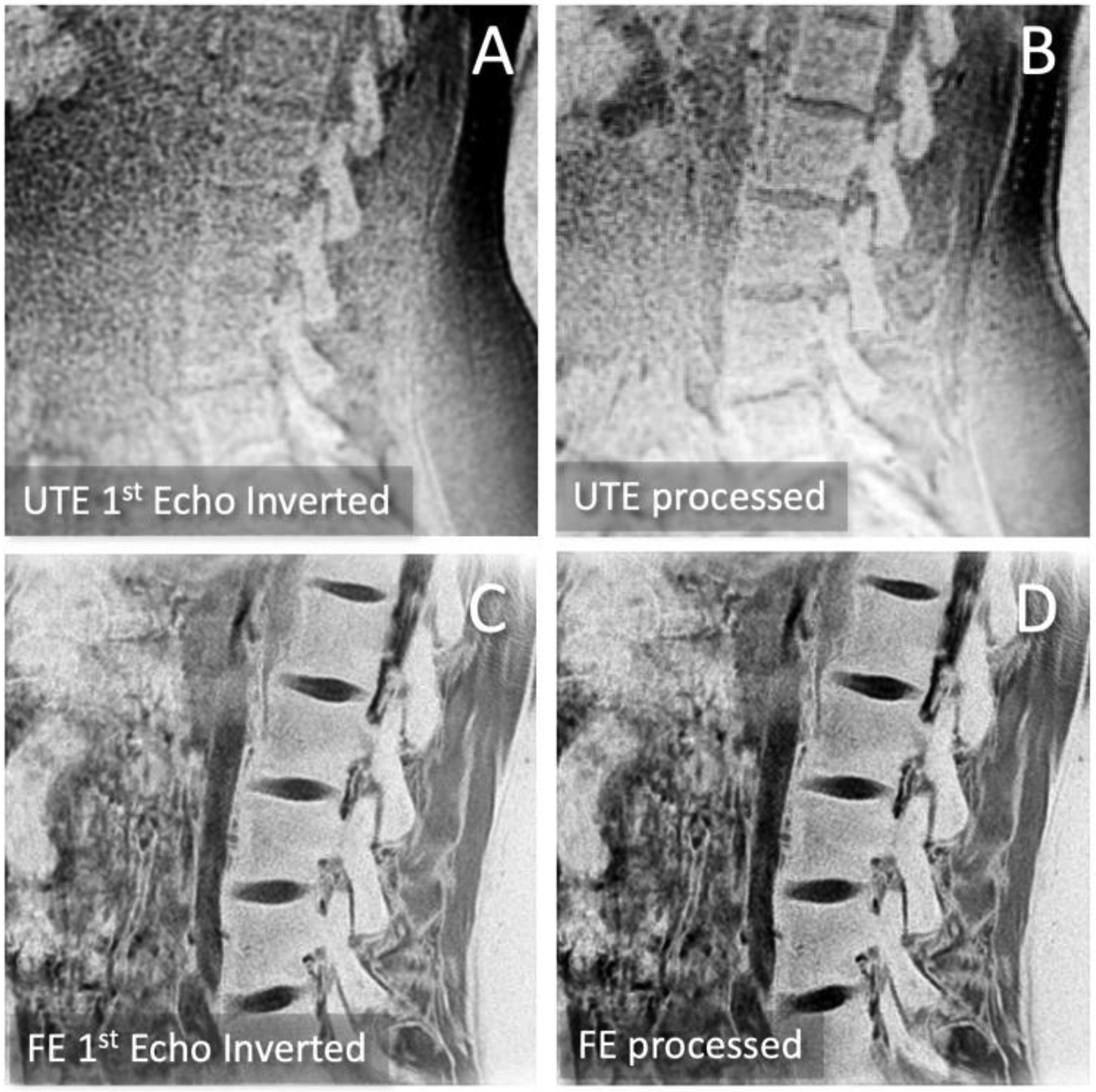
Processed images to create CT-like contrast from (A) UTE 1^st^ echo image, (B) UTE multiecho images, and (C) FE 1^st^ echo image, and (D) FE multiecho images. Differences between sequences (UTE vs. FE) and improvements in contrast and image quality from multiecho processing are apparent.

To create CT-like appearance from the bone-oriented MRIs, and to demonstrate the advantage of using multiple images from multi-echo acquisition, the images were processed in the following 2-ways, directly on the scanner console. First set of CT-like images were created from the first echo images from UTE (TE=0.1 ms, Figure 1B) and FE (TE=4 ms; Figure 1D) were simply inverted (Equation (1)) to make bone tissues bright and surrounding tissues dark (Figure 2AC).


(1) $$\text{First Echo}\;CT\text{-l}ike\;\text{Image}=\frac{1}{\text{Source Image}}$$

Second set of CT-like images were created from all images (with varying TEs) in the multi-echo series. For each sequence of UTE and FE, all of the source images from different TEs were averaged, and then inverted to yield the final CT-like images (Figure 2BD) following equation (2), where *i*=source images corresponding to each TE and *n*=number of TEs.


(2) $$\text{Multi-Echo CT-like Image}=\frac{\text{n}}{\sum_{i=1}^{n}{\text{Source Image}}_{i}}$$

### 2.4. Signal-to-Noise Ratio (SNR), Contrast-to-Noise Ratio (CNR)

**Figure 3.**
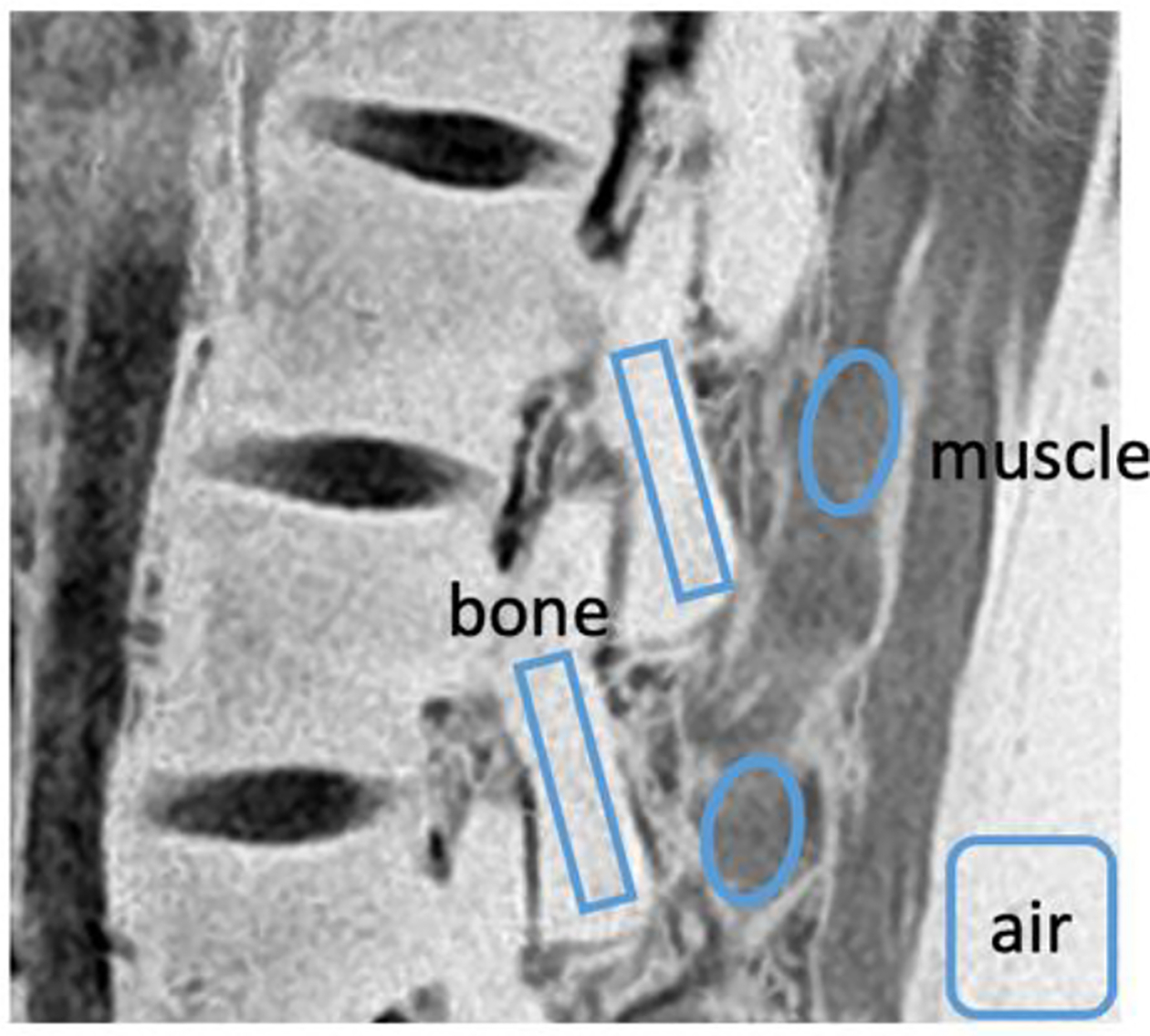
Regions of interest including bone of the pars interarticularis, paraspinal muscles, and air, analyzed to determine SNR and CNR.

Regions of interest (ROI) were drawn on a sagittal slice of the processed CT-like images that depicted cortical bone of the pars interarticularis and surrounding paraspinal muscles (Figure 3). The ROIs were placed manually by a scientist with over 10 years of experience in imaging research. ROIs for the bone were placed along the pars interarticularis, and ROIs for the muscle were placed in adjacent paraspinal muscle posterior of the bone ROI. Within each ROI, we determined the mean signal intensity (*SI*_*mean*_). ROI was also placed in the background (air) to determine the standard deviation (*SI*_*SD*_) of the noise signal intensity. We evaluated slice-to-slice variability as coefficient of variation in the mean signal intensity of bone ROI in 5 consecutive slices of two datasets, and found less than 2% variability regardless of the sequence used.

SNR was determined as the mean signal intensity divided by standard deviation of the noise in the air [22] (Equation (3)).


(3) $${SNR}^{1}=\frac{{SI}_{\text{mean}}^{1}}{{SI}_{SD}^{\text{noise}}}$$

CNR was determined as the difference in mean signal intensity between selected ROIs divided by standard deviation of the noise [23] (Equation (4)).


(4) $${CNR}^{1-2}=\frac{{SI}_{\text{mean}}^{1}{-SI}_{\text{mean}}^{2}}{{SI}_{SD}^{\text{noise}}}$$

### 3.2. Statistics

SNR and CNR values between the four types of CT-like images (processed from UTE 1st echo, UTE multi-echo, FE 1^st^ echo, and FE multi-echo) were compared using fully-facotrial two-way ANOVA [24,25] to determine the effects of the sequence (UTE vs. FE) and the use of 1^st^ echo image vs. all three images from the multi-echo. Systat statistical analysis software (v12, Grafiti LLC, Palo Alto, CA, USA) was used. The significance level was set at 5%.

### 2.6. Spondylolysis Evaluation

**Figure 4.**
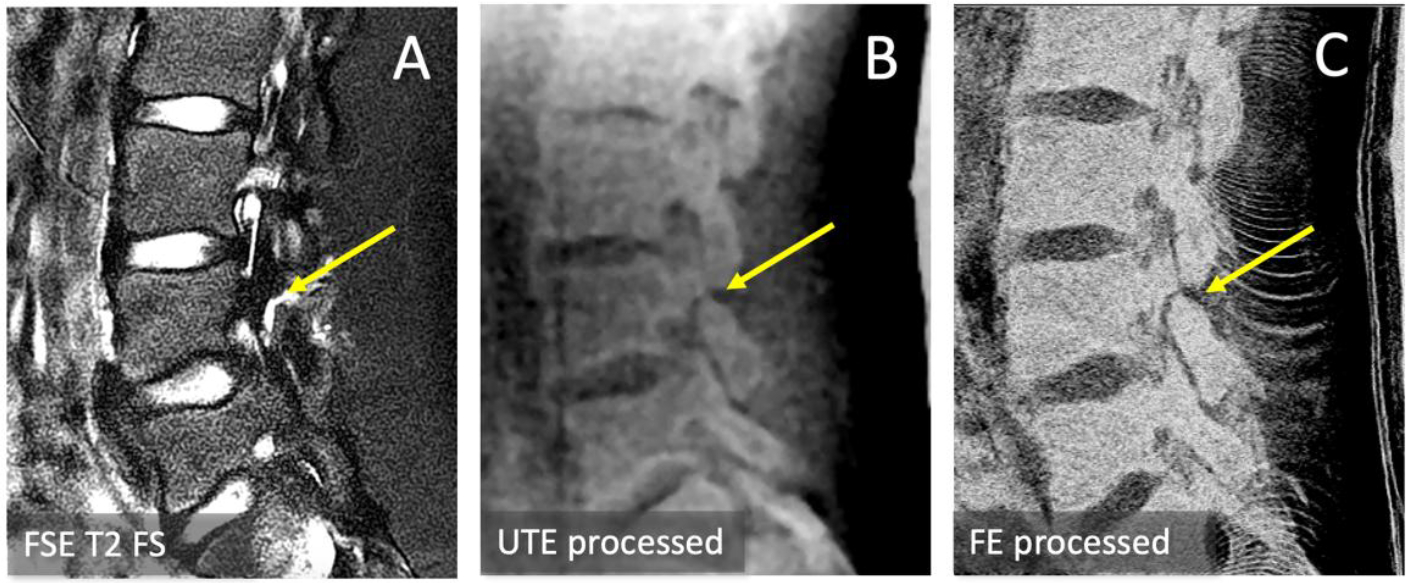
Detection of a moderately-sized pars defect (arrows) in an adolescent athlete with persistent low back pain using FSE T2 FS (A), UTE multiecho processing (B) and FE multiecho processing (C). The processed images may enable easier and more confident diagnosis of isthmic spondylolysis.

On the two subjects with low back pain, we compared conventional SE T2, multi-echo processed UTE, and multi-echo processed FE images to evaluate the presence of spondylolysis (Figure 4).

## 3. Results

### 3.1. Observations

Compared to FSE T2 (Figure 1A), CT-like processed images from 1^st^ echo (Figure 2AC) and multi-echo (Figure 2BD) UTE and FE images all depicted spinal bone distinctly with high signal intensity, similar to CT. UTE (Figure 2AB) and FE (Figure 2CB) images, while both providing CT-like contrast, had notable differences. UTE images were softer while providing a flatter contrast for the soft tissue, making it easier to distinguish the bone if the reader is unfamiliar with the anatomy. In contrast, FE images were markedly sharper and depicted bone with higher contrast. But FE images depicted many of non-bone tissues (e.g., fascia of muscle) with similarly high signal intensity as bone. This makes FE imaging less desirable for tasks such as automatic segmentation and visualization of bone, but more desirable for evaluating small features such as hairline fractures. When comparing CT-like images from 1^st^ echo (Figure 2AC) vs. multi-echo (Figure 2BD), the multi-echo images showed an improvement, with less noise and better contrast for bone overall.

### 3.2. SNR and CNR

Table 1 summarizes SNR and CNR values for the CT-like processed images. CT-like images from UTE data had mean SNR values of the bone ranging from 90 to 100, which was significantly higher (p=0.0002) than the values from the FE data (mean SNR range of 38 to 53). However, the CNR (bone-muscle) values were slightly higher (p=0.09) for FE (mean CNR ranging from 15 to 22) compared to UTE (CNR of 13 to 16). The use of multi-echo images, for both UTE and FE data, improved the CNR values significantly (p=0.03): For UTE, CNR on average improved by 2.7, while for FE, CNR improved by 6.9. This corroborates visual improvements in bone contrast observed in the image comparison in Figure 2.

### 3.3. Spondylolysis Depiction

In one of the low back pain subjects, we detected a progressive spondylolysis with a moderate-sized bone defect seen as a gap with high signal intensity in FSE T2 (Figure 4A, **arrow**), and a clear non-union in both the multi-echo processed UTE (Figure 4B, **arrow**) and multi-echo processed FE (Figure 4C, **arrow**) images. We also measured signal profile across the bone defect in UTE processed and FE processed images. Defect width measured as full width half maximum were 2.75 mm and 2.39 mm for UTE and FE, respectively.

## 4. Discussion

This study has demonstrated the feasibility of spine bone MRI to yield CT-like contrast. Through the employment of multi-echo UTE and FE sequences combined with straightforward image processing techniques, we have observed discernible enhancements in image quality and bone contrast compared to using single echo approaches. Our findings are encouraging, suggesting that these approaches may can be used to obtain CT-like images useful for evaluating spondylolysis, albeit with limitations.

Our study compared multi-echo UTE and multi-echo 3D FE sequences and demonstrated that, while both sequences can depict cortical bone reasonably well, UTE provided a flatter contrast for soft tissues that made distinguishing bone (appearing dark) from surrounding tissues (appearing bright) easier, while FE images had greater spatial resolution and bone-muscle contrast than UTE, which will be invaluable for detecting finer fractures. We also showed that processing all images from multiecho acquisition provided significantly greater CNR for bone, for both UTE and FE acquisitions.

Our study builds upon past studies with similar purpose of imaging bone using MRI. Many utilized similar approach of acquiring images that depict bone with a signal void and surrounding tissue with higher signal intensity and inverting the image to provide CT-like appearance [21,26,27]. These studies have demonstrated that these conventional sequences can indeed provide good depiction of cortical bone structure, and in one study [27], high accuracy (>90%) for detecting bone fracture was demonstrated. Our result generally corroborated these past results, and suggested benefit of using multiecho images for improving image quality. While we did not formally investigate the effect of different processing techniques, our simple averaging and inverting approach can be performed on any MRI console using built-in image processing tools. Various other processing techniques such as rescaled echo subtraction [28], simple inversion of a single echo [27], or summation of all but the last echo followed by the last echo [21] have been attempted with varying degrees of success. There are even more advanced approaches available such as using deep learning to create CT-like (pseudo-CT) images from MRI [18,29,30], but these currently require extensive off-line processing that may delay clinical evaluation.

This is an early study with many limitations. While the feasibility has been shown, a comparative study using CT reference images is needed. Since it is difficult to acquire both CT and MRI without clinical need, initial study could utilize cadaveric specimens, as done previously [18]. Also, the number of subjects used was very small but that maybe typical in early technical development. Nonetheless, a larger study will be needed in the future to confirm the present findings and to make the results generalizable for the intended application. We found that the differences in SNR and CNR between MR sequences were substantial, and the trend will likely be similar with greater number of subjects, since the signal intensity of cortical bone and muscle may not vary markedly between subjects without particular pathology. While it would have been ideal to involve radiologists to compare the image quality and the diagnostic performance for detecting pars fractures, that would require quite a large number of subjects, as well as reference CT images. This is a likely future extension of the current work. As an early study, we were able to enroll two young athletes with persistent low back pain, and positively showed spondylolysis is one of them in Figure 4, demonstrating clinical feasibility.

Despite advances in pulse sequences and post-processing techniques, there is still no MRI-based method that can produce images equivalent to CT scans. In terms of techniques appropriate for bone fracture evaluation at the pars, both techniques discussed in this paper, along with past similar approaches based on conventional image processing [19,21,27] might provide value. The use of artificial intelligence to synthesize pseudo-CT images from MRI images [18,31,32], while yielding the best bone contrast (reaching CNR of 100 to 150 [18], compared to about 20 in this study) and CT-like appearance, are not yet practical. Future work will explore possibilities of improving bone imaging using more advanced image processing and/or deep learning techniques.

## 5. Conclusions

In conclusion, this study demonstrated feasibility of two MRI techniques to provide a radiation-free alternative for the evaluation of spondylolysis, with clinical application shown on a young subject with pars defect. Further advancements in MRI technology with multi-echo UTE and FE have the potential to transform the standard of care for adolescent athletes and others with isthmic spondylolysis, offering a safer, equally effective, and radiation-free imaging alternative.

## Figures and Tables

**Table 1.** (Top) Mean and standard deviation of SNR and CNR values from various regions of interest. (Bottom) p-values from two-way ANOVA to assess the effects of sequence (UTE vs. FE) and processing (1^st^ echo vs. multi-echo) on SNR and CNR.

	Mean (+/− Std. Dev.) Values for Each Sequence			
Measurement	UTE 1st echo	UTE multi	FE 1st echo	FE multi
Bone SNR	107 (66)	89.8 (29.5)	38.1 (10.3)	53.3 (16.3)
Muscle SNR	94.4 (65.4)	74.0 (32.8)	23.4 (7.0)	31.9 (14.2)
Bone-Muscle CNR	13.0 (5.2)	15.7 (5.0)	14.6 (4.2)	21.5 (8.5)
	Two-Way ANOVA: Effect of			
Measurement	UTE vs FE	1 vs. multi	Interaction	
Bone SNR	**0.0004**	0.9289	0.2235	
Muscle SNR	**0.0002**	0.6556	0.2864	
Bone-Muscle CNR	0.0935	**0.0311**	0.3327	

## Data Availability

The data that support the findings of this study are not publicly available due to reasons of sensitivity. Anonymized data may be available from the corresponding author upon the review of the request. Data are located in controlled access data storage at the corresponding author’s institution.
